# Shorter telomere length is associated with COVID-19 hospitalization and with persistence of radiographic lung abnormalities

**DOI:** 10.1186/s12979-022-00294-9

**Published:** 2022-08-22

**Authors:** Miriam Retuerto, Ana Lledó, Beatriz Fernandez-Varas, Rosa Guerrero-López, Alicia Usategui, Antonio Lalueza, Rocío García-García, Esther Mancebo, Estela Paz-Artal, Leandro Sastre, Rosario Perona, José L. Pablos

**Affiliations:** 1 Servicio de Reumatología, Instituto de Investigación Hospital 12 de Octubre, 28041 Madrid, Spain; 2 Servicio de Telomeropatías, Instituto de Investigaciones Biomédicas CSIC/UAM; CIBER enfermedades raras; Instituto de Investigación Hospital Universitario La Paz, Madrid, Spain; 3 Servicio de Medicina Interna, Instituto de Investigación Hospital 12 de Octubre, Madrid, Spain; 4 Universidad Complutense de Madrid, Madrid, Spain; 5 Servicio de Neumología, Instituto de Investigación Hospital 12 de Octubre, Madrid, Spain; 6 Servicio de Inmunología, Instituto de Investigación Hospital 12 de Octubre, Madrid, Spain; 7 Instituto de Salud Carlos III, Madrid, Spain

**Keywords:** SARS-CoV-2, COVID-19, Telomere length, Pulmonary fibrosis

## Abstract

**Background:**

Age and comorbidity are the main determinants of COVID-19 outcome. Shorter leukocyte telomere length (TL), a hallmark of biological aging, has been associated with worse COVID-19 outcomes. We sought to determine TL in patients with severe COVID-19 requiring hospitalization to analyze whether clinical outcomes and post-COVID-19 manifestations are associated with shorter TL.

**Results:**

We analyzed 251 patients with PCR-confirmed COVID-19, hospitalized in the first months of the pandemics. We determined TL in PBL at admission by quantitative-PCR (qPCR) analysis in patients. A healthy cohort from the same area with a similar age range (*n* = 169) was used to calculate TL Z-scores. After hospital discharge, 144 COVID-19 survivors were followed-up for persistent COVID-19 manifestations. A second TL determination was performed in a smaller group of 63 patients 1 year later and compared with baseline TL.

Hospitalized COVID-19 patients had a decreased baseline age-adjusted TL Z-score compared to the reference group. No differences in Z-scores were observed in patients with different COVID-19 outcomes, classified as WHO ordinal scores. In 144 patients, followed for a median of 8 months, post-COVID manifestations were not associated to differences in TL. Persistence of lung radiographic abnormalities was associated with shorter baseline TL. In patients with a second TL determination, further telomere shortening (TS) was observed in 35% and telomere lengthening in 49%. Patients with further TS had suffered a more severe disease.

**Conclusion:**

Shorter TL is associated with COVID-19 hospitalization but not with hospital clinical outcomes nor with persistent post-COVID-19 manifestations. Delayed resolution of radiographic lung abnormalities was also associated with shorter TL.

## Background

The clinical outcomes of SARS-CoV-2 infection are highly heterogeneous, from asymptomatic to respiratory failure and death [1, 2]. At the first peak of the coronavirus disease 19 (COVID-19) pandemics in March–April 2020 in Spain, hospitalization was mainly indicated for patients with severe disease, characterized in most cases by radiographic lung infiltrates and respiratory failure requiring oxygen therapy [3]. In hospitalized COVID-19 patients, a high mortality occurred in the earliest case series, and the strongest risk factor associated with severe disease and death was older age [3, 4].

Previous comorbidities such as obesity and cardiometabolic diseases, are also age-independently associated with worse COVID-19 outcomes [1–4]. Obesity and cardiovascular morbidities are associated with premature aging, as marked by accelerated telomere shortening [5, 6]. Among aging related pathogenicity factors involved in severe lung COVID-19, immunosenescence, and excessive inflammatory damage with impaired regeneration of lung tissues have been identified [7–9]. Virus induced cell senescence has also been shown to significantly contribute to COVID-19 pathology which can be attenuated by senolytics in experimental models [10, 11]. In addition, the expression of COVID-19 receptor ACE2 increases upon telomere damage in cellular and animal experimental models therefore facilitating cell infection [12].

Previous studies have reported an association between shorter TL and different measures of COVID-19 severity in different populations [13–15]. Genetic or acquired telomere shortening has also been identified as risk factors of idiopathic pulmonary fibrosis (IPF) [16, 17]. The mechanistic link between shorter TL and lung fibrosis relates to the reduced capacity of respiratory epithelial cells with TS to recover from damage, resulting in enhanced connective tissue repair and fibrosis [18]. Short telomeres in lung epithelial cells make them more susceptible to different noxae and reduce their regenerative capacity after oxidative damage [19]. Shorter TL has also been found associated with worse survival in other respiratory conditions such as ARDS in sepsis [20]. Therefore, the progression of lung lesions during acute COVID-19 of its resolution may be conditioned by TL. A study in a small group of post-COVID-19 patients suggests that persistent lung fibrotic features were also associated with shorter telomeres [21].

We here analyzed TL in a cohort of severe hospitalized COVID-19 patients to confirm associations with the different outcomes during acute disease and along further follow-up in survivors.

## Results

### Telomere length in COVID-19 patients and healthy controls

**Fig. 1:**
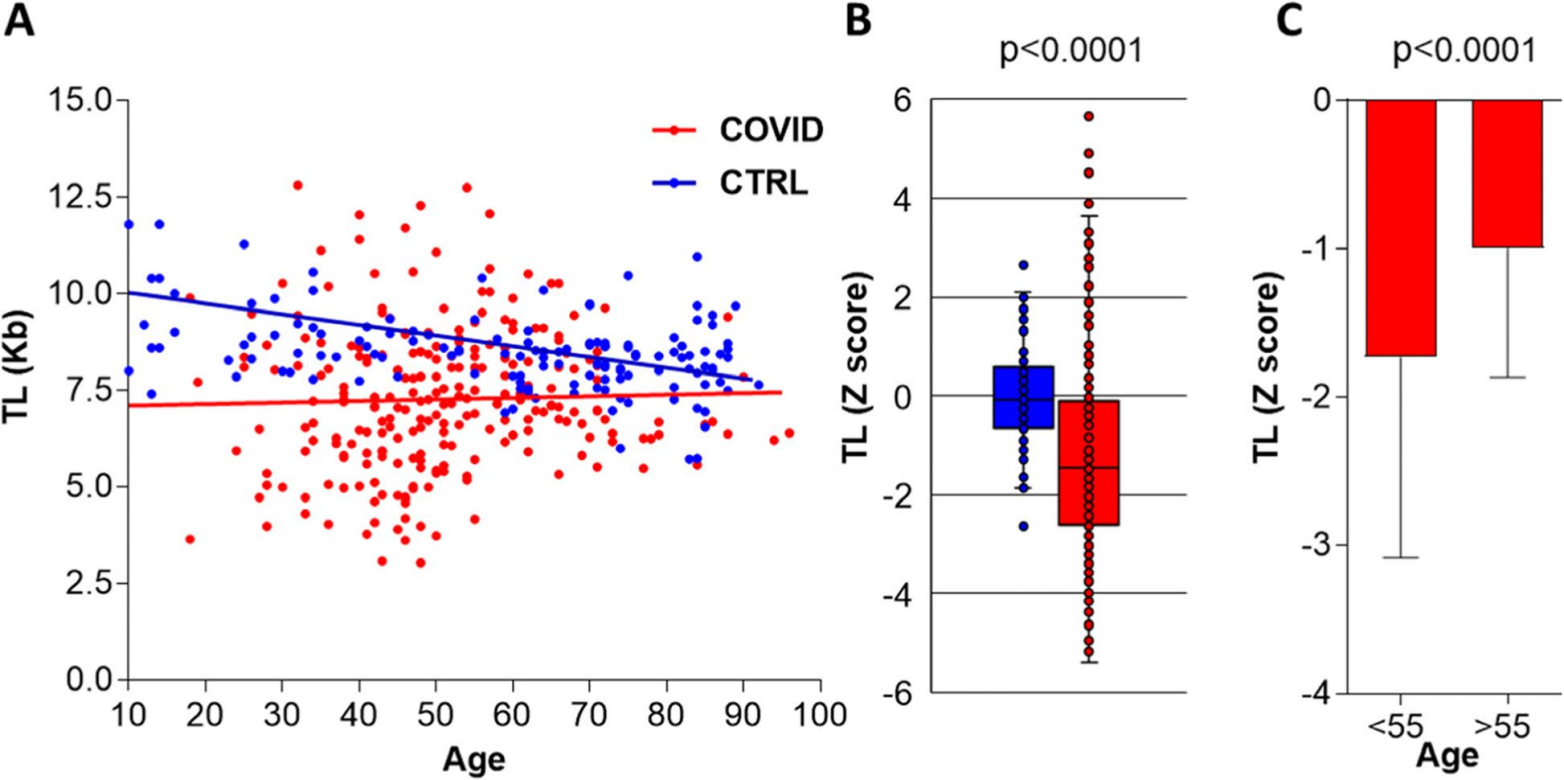
Telomere length in hospitalized COVID-19 patients and healthy controls. **A** TL in Kb as determined by PCR in healthy controls (CTRL; blue, *n* = 169) and COVID-19 patients (red, *n* = 251) plotted against age. TL/age correlation coefficient by Spearman rank test: COVID-19 *r* = 0.1 *p* = 0.10, CTRL *r* = 0.46 *p* < 0.0001. **B** TL Z-score of healthy controls (blue) and COVID-19 patients (red). Median and IQR [25, 75%] are represented. **C** TL Z-score in COVID-19 patients by age groups, younger or older than 55 years-old

In the healthy controls group (HC), TL distribution showed a significant inverse correlation with age (*r* = 0.46, *p* < 0.0001) as expected (Fig. 1A). In contrast, a higher dispersion and no correlation between age and TL were observed in the hospitalized COVID-19 cohort (*r* = 0.1, *p* = 0.1). Hospitalized COVID-19 patients had a significantly decreased age-adjusted TL Z-score compared to HC (− 1.45 [− 2.64, − 0.12] vs − 0.19 [− 0.66, 0.44]; *p* < 0.0001) (Fig. 1B). This reduced, age-adjusted TL occurred in all age groups, but it was significantly lower in patients under 55 years old (− 1.73 [− 3.09, − 0.30]) compared to older patients (− 1.02 [− 1.95, − 0.51]; *p* < 0.0001) (Fig. 1C).

**Fig. 2:**
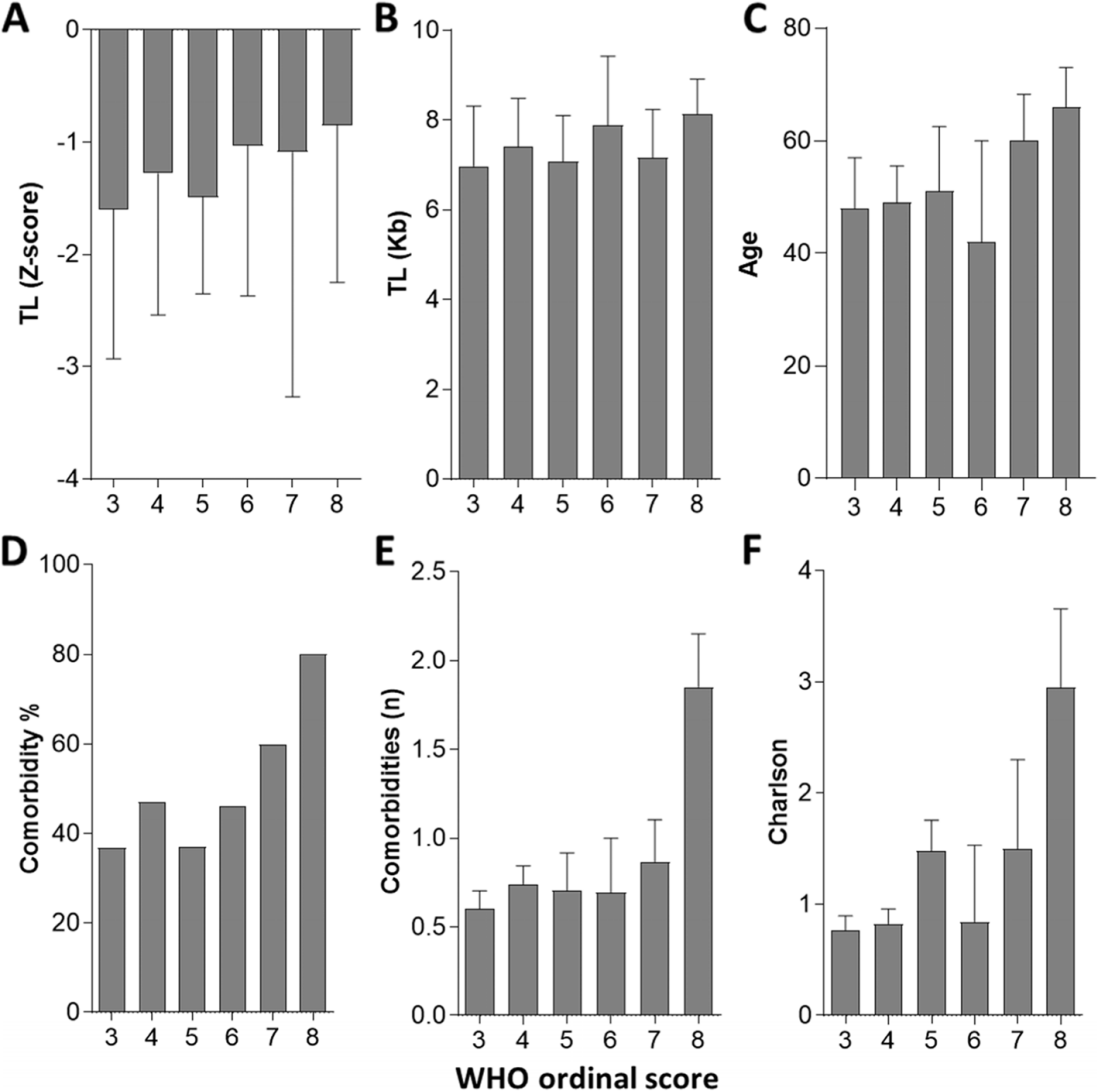
Telomere length, age, and comorbidity in hospitalized COVID-19 patients by severity (ordinal WHO score). **A** Median TL (Z-score) by ordinal severity WHO scores. **B** Median absolute TL (Kb) by ordinal severity WHO scores. **C** Median age by ordinal severity WHO scores. **D** Percentage of patients with at least one comorbidity by ordinal severity WHO scores. **E** Median number of comorbidities by ordinal severity WHO scores. **F** Median Charlson index by ordinal severity WHO scores

Age-adjusted TL Z-score in patients with severity scores 3 (hospitalized patients without oxygen therapy) to 8 (death) according to the WHO ordinal scale is shown in Fig. 2. Either age-adjusted or absolute (Kb) TL was similarly decreased in all the groups by COVID-19 severity scores (ANOVA *p* = 0.6) (Fig. 2). Another relevant measure of COVID-19 severity was the duration of oxygen therapy which did neither correlate with age-adjusted TL (*r* = − 0.03, *p* = 0.72). We also analyzed whether several biomarkers of the inflammatory response or tissue damage such as the plasma levels of CRP, ferritin, LDH or D-dimers, also associated to severity of COVID-19 were correlated with TL. No correlation between TL Z-score with any of these markers was found (data not shown), further supporting that the progression to more severe disease is not associated with shorter age-adjusted TL in this cohort of hospitalized patients.

Since this lack of association was somewhat unexpected, we analyzed whether well stablished predictors of severe disease such as age, sex, and comorbidity were associated with severity in this cohort. As expected, the median age was different by severity scores, particularly in the more severe 6–8 groups (58 [42] years old) compared to the milder 3–5 groups (49 [43]; *p* = 0.04) (Fig. 2). The presence of comorbidity, defined as at least one previous selected comorbid condition as defined in Table 1, was also increased according to the severity score, such that in the 6–8 score group, it was significantly higher compared to 3–5 score group (65% vs 41%, *p* = 0.03). The median Charlson comorbidity index as well as the median number of selected comorbidities were also different by severity scores, 0 [0,1] in class 3 vs 2 [1, 3] in class 8 groups (*p* = 0.001) and 0 [0,1] in class 3 vs 3 [0,5] in class 8 groups (*p* = 0.0005) respectively (Fig. 2). We also found a higher proportion of male than female patients in the more severe 6–8 groups (23,5% vs 12,7%, *p* = 0.03). COVID females and controls had also shorter adjusted TL than males (− 1,6 [− 2,7, − 0,1] vs − 1,1 [− 2,7, − 0,1], but sex adjusted Z scores were similarly decreased in all the groups by COVID-19 severity scores in either male or female patients.

**Table 1:** Demographic and clinical characteristics of hospitalized COVID-19 patients and long-term evolution after discharge

Variables	***n*** = 251
Age	49 [7–96]^a^
Gender, female	102 (40.6)
Any Comorbidity	98 (39)
Obesity	61 (24.8)
Hypertension	53 (21.2)
Cardiovascular disease^b^	13 (5.2)
Respiratory disease^c^	25 (10)
Cancer	15 (6)
Dementia	4 (1.6)
Number of comorbidities	0 [0–1]
Charlson Comorbidity index	0 [0–2]
Radiographic pneumonia	237 (94.4)
Laboratory tests	
C reactive protein (mg/dL)	9 [0.05–60.4]
Neutrophils (cells/μL)	5.100 [900–14.800]
Lymphocytes (cells/μL)	1.000 [200–4.700]
D-dimer (μg/L)	711 [172–9.300]
Ferritin (μg/L)	906 [7.9–11.410]
WHO Ordinal Scale^d^	
3	95 (37.8)
4	81 (32.3)
5	27 (10.8)
6	13 (5.2)
7	15 (5.9)
8	21 (8.4)
COVID-19 Therapy	
Remdesivir	6 (2.4)
Tocilizumab	68 (27)
Anakinra	4 (1.6)
Steroids	106 (42.6)
Post-COVID manifestations^e^	
Persistent symptoms	97 (67.4)
Systemic	70 (48.6)
Dyspnea	65 (45.1)
Musculoskeletal	10 (6.9)
Neuro-psychiatric	44 (30.6)
Dermatological	9 (6.6)
Genitourinary	3 (2.1)
Persistent radiographic abnormalities	54 (48.6)^f^
Persistent respiratory failure	9 (6.3)

^a^Values represent n (%) or median [IQR]

^b^Heart attack, stroke, peripheral arterial disease

^c^Asthma, COPD, OSAHS, lung hypertension

^d^World Health Organization (WHO) Scale: 0–2 ambulatory; 3 Hospitalized no oxygen therapy; 4 Oxygen by mask or nasal prongs; 5 Non-invasive ventilation or high-flow oxygen; 6 Intubation and mechanical ventilation; 7 Ventilation and additional organ support; 8 Death

^e^Persistent clinical signs and symptoms after COVID-19 for more than 12 weeks (Median follow-up 231[132, 277] days

^f^Radiographic control was performed at 3 or more months after discharge in 166 patients

During acute COVID-19, changes in the distribution of PBL with a profound lymphopenia and an increased neutrophil/lymphocyte ratio correlate with the severity and other markers of the inflammatory response [2, 22]. Since this factor might influence the distribution of TL in the whole PBL cell population [23], we analyzed the potential correlation between the absolute number of neutrophils or lymphocytes per ml, or the neutrophil/lymphocyte ratio, with the age-adjusted TL Z-score. Correlation between these parameters with TL were not found (data not shown).

### Telomere length and long-term evolution of COVID-19

To investigate whether TL may influence the persistence of clinical manifestations, defined as post-COVID, we analyzed persistent clinical manifestations in the subgroup of patients undergoing protocolized follow-up in our multidisciplinary ambulatory COVID-19 unit for at least 3 months after discharge (*n* = 144). These patients were followed for a median time of 231 [132, 377] days. Ninety seven out of 144 patients (67%) had persistent symptoms (Table 1). TL was similar in patients with or without post-COVID symptoms, Z-score − 1.31 [− 2.7, − 0.15] vs − 1.60 [− 2.72, − 0.17] respectively (*p* = 0.57).

Most prevalent symptoms were respiratory, mainly persistent dyspnea in 65 patients (45%), but only 9 patients (6%) required ambulatory oxygen therapy after 3 months. Age-adjusted TL was similar in patients with persistent dyspnea compared to patients without persistent dyspnea (data not shown).

**Fig. 3:**
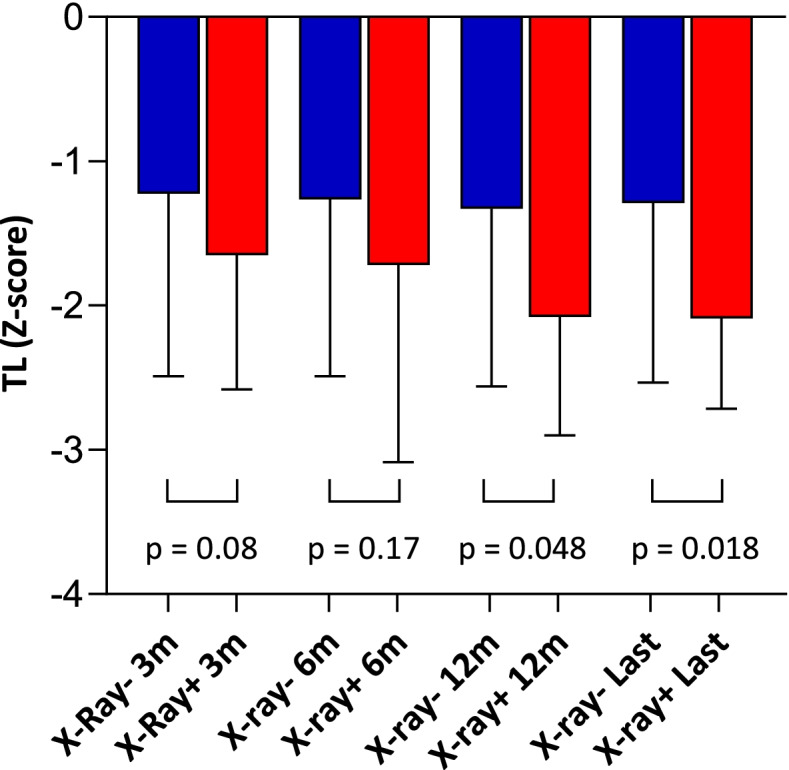
Comparative Telomere Length in hospitalized COVID-19 patients by persistence or resolution of lung radiographic abnormalities. Median TL (Z-score) in patients with (+) red, or without (−) blue, lung radiographic abnormalities at each time point. Number of patients with radiographic abnormalities at each time point was 54/163 (3 months), 32/165 (6 months), 29/166 (12 months) and 26/164 (last follow-up)

In 178 patients, thoracic radiological follow-up after 3 months from discharge was performed. Median of last radiological follow-up was 231 [131, 377] days. We analyzed in all patients with available thoracic X-ray studies, whether age-adjusted TL was associated with persistence of radiographic lung abnormalities, including lung infiltrates or fibrotic features. The proportion of patients with radiographic abnormalities decreased along follow-up such that 33, 19, 17 and 16% had abnormalities at 3, 6, 12 months or last follow-up respectively. Patients with persistent abnormalities had shorter TL than patients with complete resolution of radiographic infiltrates. In patients with persistent abnormalities at 12 months or longer follow-up the difference was statistically significant (Fig. 3), with a post-hoc statistical power of the available study sample at 12 months of 88% for a confidence level of 95%.

### Changes in telomere length after COVID-19 recovery

**Fig. 4:**
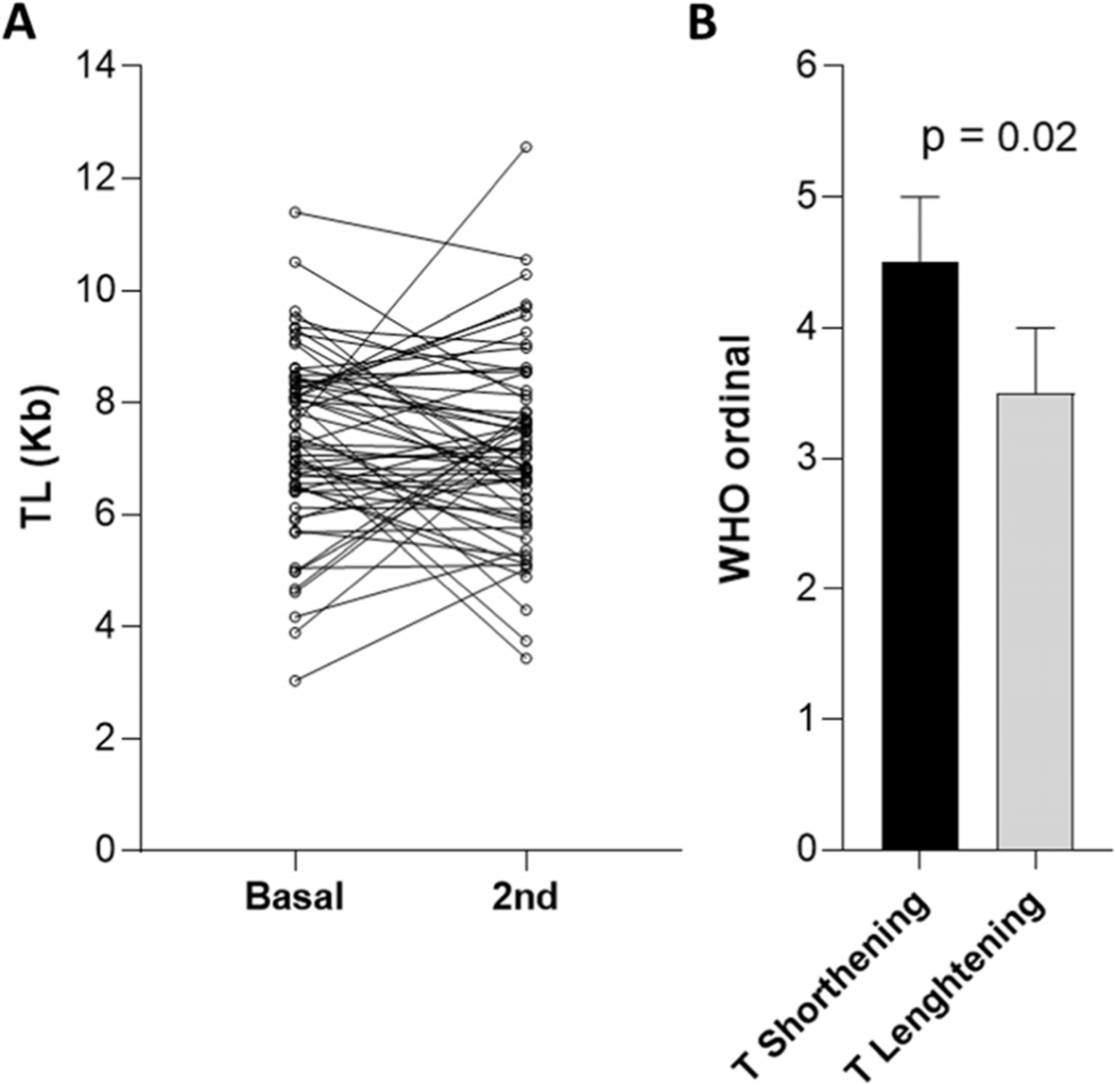
Changes in Telomere Length after 1 year of follow-up. **A** TL (Kb) change between the first determination at admission and a second determination at a median time of 425 [415, 553] days of follow-up. **B** WHO ordinal scores in patients with telomere shortening or lengthening (> 0.1 Kb) in the second sample compared with the first

In 63 patients, a second determination or TL was obtained a median time of 425 [415, 553] days after the first determination. Compared to the first determination, telomere shortening (TS) occurred in 22 patients (35%), telomere lengthening in 31 (49%), and no changes in TL in 9 (14%) (Fig. 4). We analyzed whether these changes were associated to the severity of COVID-19. Patients with further TS had a higher severity score than patients with telomere lengthening in the second determination (Fig. 4). A higher proportion of patients with TS (47%) compared with those with telomere lengthening (21%) had persistent radiographic abnormalities at 3 months (*p* = 0.04) but not at further time points. Additional severity parameters such as duration of oxygen therapy and lower lymphocyte counts at admission showed a similar but non-significant trend. Age was similar in both groups of patients (data not shown).

Since differences in TL between myeloid and lymphoid leukocytes have been described [23], we analyzed whether the change of the PMN/lymphocyte ratio correlated with the absolute change in TL (Kb) in the second compared to the first blood sample. No correlation between the change in either leukocyte population or their ratio and the TL change was observed.

## Discussion

Our data are consistent with previous observations showing age adjusted shortened TL in patients with severe COVID-19 requiring hospitalization, compared with healthy individuals [15]. However, we did not detect an association between TL and more severe outcomes such as invasive ventilation or death in hospitalized patients as suggested by some studies [13, 14]. Methodological differences such as the sample size and reference population, the inclusion or not of milder patients, and the variable definitions of severity may explain the different conclusions. The largest study performed in the UK biobank showed differences in Z-score standardized TL assessed several years prior to SARS CoV-2 infection, between hospitalized and non-hospitalized COVID-19 community cases [15]. Although we did not include community cases, the observed difference in TL between healthy controls and hospitalized patients is consistent with the results of the UK study. In our series as well as in the UK study, other known severity factors, such as sex, chronological aging and comorbidities, seem more relevant for the progression of respiratory failure and do not support the value of TL as prognostic factor in hospitalized patients. The pathogenesis of severe COVID-19 includes an initial phase dependent on innate immune responses and a later phase more dependent on inflammatory damage [24, 25]. Shorter TL might be more relevant in the initial phase of the disease increasing the chance of hospitalization, independently of later complications that determine the final outcome. Nevertheless, whether the association between TL and COVID-19 hospitalization reflects causality requires further studies.

A relevant outcome of COVID-19 evolution relates to the persistence of certain clinical manifestations after recovery from acute disease, most importantly respiratory dysfunction, but also a variety of manifestations termed post-COVID-19 [26, 27]. In our series, a high proportion of patients show persistent lung radiological abnormalities at 6 and 12 months after acute disease whereas the proportion of respiratory functional impairment is much lower. We find that delayed radiographic resolution but no other post-COVID-19 manifestations in survivors is associated with shorter telomeres. Idiopathic pulmonary fibrosis (IPF) is the best characterized clinical consequence of genetic or acquired TS and in experimental models, it relates to enhanced epithelial damage after different insults [18, 19]. Although COVID-19 may lead to lung fibrotic changes after the acute phase, lung function tends to improve and a progressive fibrosis similar to IPF is not observed [28]. A previous study of a small cohort evaluated specifically fibrotic features in CT scans at 4 months after acute COVID-19 and found an association between TL and fibrotic changes [21]. Our data are consistent with slower resolution of lung damage in patients with shorter TL. However, since we performed radiographic but not CT scan follow-up in most patients, variation on fibrotic features could not be specifically evaluated.

Changes in TL of different sign have been previously observed in different settings. In critically ill patients, short term TS has been associated with younger age and more severe disease in the acute setting [29]. In the long-term, telomere lengthening has been observed after therapeutic interventions in obese patients [30]. Our data show that TS or lengthening variably occurs after COVID-19 recovery. TS was associated with severity of COVID-19 during hospitalization, pointing to either reduced telomere recovery or enhanced telomere damage in patients with more severe acute disease. However, this data is limited by the smaller number of patients with follow-up samples and the potential variability of different qPCR determinations of TL and therefore, further studies are warranted to further explore the significance of these changes in TL and its long-term clinical significance after recovery.

## Conclusions

Our data support previous observations on the role of baseline TL as a risk factor for hospitalization in COVID-19, but not for in hospital complications nor persistent post-COVID-19 manifestations that occurred independently of TL.

We observed an association between delayed radiographic resolution and shorter baseline TL that is consistent with the proposed role of TS in lung repair after damage.

## Methods

### Telomere length analysis

We determined TL in DNA from unfractionated peripheral blood leukocytes (PBL) obtained at hospital admission from a COVID-19 hospitalized cohort (*n* = 251) by quantitative-PCR (qPCR) analysis as previously described [31]. DNA was extracted by NZY gDNA isolation Kit (NZTech, Lisbon, Portugal). Included COVID-19 patients had been admitted in Hospital 12 de Octubre, a tertiary hospital in Madrid (Spain), at the first peak of the pandemics, from March 14th to May 1st of 2020. In all cases, a microbiological diagnosis (PCR) was obtained.

TL was also determined in a healthy control group from the same area, with a similar age and sex distribution, obtained before the COVID-19 pandemics (*n* = 169), to establish a TL standard curve and to obtain a Z-score measure of the deviation of TL of individual patients from the age standardized curve. The Z-score compared the TL value of each individual with the age-matched mean and standard deviation (SD) of the values obtained in the controls: (individual’s value – population mean)/population SD, age-matched population within 9 years on average.

To evaluate the potential long-term impact of COVID-19 on TL, patients were contacted 1 year after hospitalization and, in those willing to collaborate, a blood sample for a second TL determination was obtained.

Severity of COVID-19 was classified according to the WHO ordinal scoring system, as follows: (3) hospitalized, not requiring supplemental oxygen; (4) hospitalized on oxygen via mask or nasal prongs; (5) hospitalized, on noninvasive ventilation or high-flow oxygen; (6) hospitalized, intubated and on mechanical ventilation (MV); (7) hospitalized on MV and additional organ support (renal replacement therapy, vasoactive drugs or extracorporeal membrane oxygenation), and (8) dead (https://www.who.int/publications/i/item/covid-19-therapeutic-trial-synopsis).

In survivors undergoing protocolized ambulatory follow-up (*n* = 144), persistent manifestations after 3 months were registered according to WHO post-COVID clinical definitions (https://www.who.int/publications/i/item/WHO-2019-nCoV-Post_COVID-19_condition-Clinical_case_definition-2021.1). COVID-19 patient characteristics are summarized in Table 1.

### Statistical analyses

Statistical analyses were performed with STATA/IC version 14.0 (Stata Corp) and GraphPad Prism 5 (GraphPad, Inc). Quantitative data are shown as the median with interquartile range [IQR], whereas qualitative variables were expressed as absolute or relative frequencies. Absolute (Kb) or age-adjusted (Z-score) TL was compared in the different groups by non-parametric Mann Whitney or ANOVA (Kruskal-Wallis) tests. χ^2^ or Fisher’s exact tests were used for categorical variables as appropriate. Correlation between continuous variables was calculated by Spearman’s rank correlation. Regarding the primary objective, the statistical power of the available study sample (COVID group, *n* = 251; reference group, *n* = 169), based on estimates obtained from our previous studies [31, 32], was 99% for a confidence level of 95%.

## Data Availability

Data generated or analyzed during this study are included in this published article. Data not shown are available from the corresponding author on reasonable request.

## Abbreviations

TS: Telomere shortening
TL: Telomere length
qPCR: Quantitative-PCR
WHO: World Health Organization
IPF: Idiopathic pulmonary fibrosis
ARDS: Acute respiratory distress syndrome
HC: Healthy controls group
PBL: Peripheral blood lymphocyte
PMN: Polymorphonuclear neutrophils
